# Detecting common allergens in dogs with atopic dermatitis in South Korean Provinces using a serological immunoglobulin E-specific allergen test

**DOI:** 10.14202/vetworld.2022.1996-2003

**Published:** 2022-08-20

**Authors:** Gareeballah Osman Adam, Yang-Gyu Park, Jeong-Hwi Cho, Jinyoung Choi, Hong-Geun Oh

**Affiliations:** 1 Department of Veterinary Medicine and Surgery, College of Veterinary Medicine, Sudan University of Science and Technology, Hilat Kuku, Khartoum 11311, Sudan; 2 Integrated Omics Institute, Wonkwang University, Iksan, Jeonbuk 54538, Korea; 3 Allergy Test Service Center, HUVET Co. Ltd., Iksan-si 54531, Korea

**Keywords:** canine atopic dermatitis, dairy, immunoglobulin E, mite, mold, serum test

## Abstract

**Background and Aim:**

Canine atopic dermatitis (CAD) is a hereditary susceptibility to the development of allergic symptoms in response to repeated exposure to generally innocuous substances known as “allergens.” Allergens can be plants, animals, mold, mites, or milk. At present, serological enzyme-linked immunoassay (ELISA) kits are used for immunoglobulin E (IgE)-specific allergen detection due to their simplicity and accuracy. This study aimed to detect allergens in dogs with CAD and determine how they differ according to season, breed, age, and sex using a serological test in six provinces in South Korea for 12 months. This will allow practitioners to easily understand the risk factors related to CAD.

**Materials and Methods:**

In this study, IgE allergen-specific ELISA kits were used. The allergens were detected in serum samples collected from different regions considering season, sex, breed, and age. Allergens were divided into the following Ten categories: 1. Dairy, yeast, and egg, 2. grains, 3. vegetables, 4. meat, 5. seafood, 6. animals, 7. mold, 8. insects, 9. mites, and 10. trees.

**Results:**

The percentage of allergens detected in males (54.8%) was higher than that of females (45.2%); 54.2% of allergens occurred in 3-year-old dogs or older. Moreover, regarding frequency, 65.6% of overall allergens occur during autumn; Chungcheongnam-do and Jeollabuk-do showed 20.7% and 20.9%, respectively. Additionally, among allergens categories, notable allergen occurrence was as follows: 38.3% corn; 28.7% potatoes; 22.7% duck; 24.4%,codfish; 31.2% animal wool; 95.6% *Aspergillus fumigatus*; 31.9% flea; 41.8% oak; and 25.0% sheep’s sorrel grass.

**Conclusion:**

This study showcases the frequency of 60 allergens in six provinces detected in dogs with CAD; most likely from food or the environment using serological ELISA kits. Environmental sensitizer results can be considered for humans suffering from allergies to avoid a similar environment. A large-scale study can be performed to evaluate the allergens in the state. However, neither a skin test nor feed analysis was conducted, which is a limitation of this study.

## Introduction

Canine atopic dermatitis (CAD) is an immunoinflammatory cutaneous disease that causes itching (pruritis), swollen spots, and elevated dark gray patches on the paws, neck, ear, mouth, tail, armpit, thighs, and other sites [1, 2]. Several owners observed that dogs with CAD start rubbing against objects, scratching, and biting their feet [3]. The etiology of CAD is not fully understood. The condition was assumed to be caused by interactions between the animal’s immune system, genetic preparedness (susceptibility), and dietary or environmental allergen exposure [4, 5]. CAD can be caused by a variety of allergens, including mites such as *Dermatophagoides pteronyssinus*, molds such as *Aspergillus fumigatus*, and a number of trees and grass, meat, and dairy products [6, 7]. Approximately 20% of worldwide domestic dogs are affected by CAD [8].

Canine atopic dermatitis diagnosis and treatment remain challenging due to the lack of specialized laboratory tests and medications [9]. Thus, doctors employ varying strategies to treat the condition, such as prescribing corticosteroids, emollients, antihistamines, antibiotics, and immunomodulatory drugs [10]. The diagnosis of CAD relies solely on clinical signs and history of exposure to some allergens, such as those mentioned above [6, 11]. In fact, the production of immunoglobulin E (IgE) antibodies (Abs) by plasma cells is triggered by allergen exposure [12]. In CAD, IgE antibodies are inevitable mediators of hypersensitivity responses [13]. Although higher unspecific IgE levels in the serum was less use due to cross-reactivity with other disorders, detection of IgE-specific allergens through IgE-specific allergen kits is important if CAD has already been clinically diagnosed to determine which allergens caused the aberrations on the skin. Studies in Korea showed that the prevalence of CAD was 8.5%, which was the second most common medical problem reported in the clinics [14, 15]. These studies, however, are either old or did not show particular allergens among dogs with CAD.

Therefore, this study aimed to screen 60 allergens in six South Korean provinces using an IgE allergen-specific enzyme-linked immunoassay (ELISA) kit to determine which allergens are most often linked to CAD and their frequency according to season, sex, breed, and allergen type. This might allow the general practitioner to easily guide the owners regarding risk factor avoidance to minimize CAD severity.

## Materials and Methods

### Ethical approval

Ethical approval was not required for this study because serum samples were used. However, samples were collected as per the standard collection procedure without any harm to dogs.

### Study period and location

This study was conducted from January to December of 2021 to cover the four seasons of the year. The seasons of the year were considered according to previously published data based on meteorological calculation [16] namely, winter (December 1–February 28), spring (March 1–May 31), summer (June 1–August 31), and autumn (September 1–November 30). All samples were examined at the Allergy Test Service Center in Huvet Corp., Iksan-si, South Korea. Dogs raised in the following provinces were tested: Chungcheongbuk-do, Chungcheongnam-do, Jeollabuk-do, Jeollanam-do, Gyeongsangbuk-do, and Gyeongsangnam-do.

### Animals

In this study, 189 client-owned dogs (both sexes) were adopted, ranging in age from months to over 3 years. Maltese, Bichon Frise, Poodle, Pomeranian, Mix, Shih Tzu, Yorkshire Terrier, Shiba Inu, Pug, French Bulldog, Dachshund, Spaniel, Golden Retriever, Welsh Corgi, Chihuahua, Husky, Spitz, and Italian Greyhound were among the 20 distinct dog breeds used. Table-1 shows the number and proportion of dogs belonging to each breed. Animals included in this study satisfied at least five of Favrot’s criteria [6, 16].

**Table-1:** Breed of animals tested.

Dog’s breed	Number	Percentage
Maltese	61	35.3
Bichon Frise	32	18.4
Poodle	26	14.9
Pomeranian	16	9.1
Mix	12	7.2
Shih Tzu	8	4.7
Yorkshire Terrier	6	3.5
Shiba Inu	4	2.3
Pug	4	2.3
French Bulldog	4	2.3
Dachshund	4	2.3
Spaniel	3	1.8
Golden Retriever	2	1.2
Welsh Corgi	1	0.6
Chihuahua	1	0.6
Husky	1	0.6
Spitz	1	0.6
Italian Greyhound	1	0.6

### Sample analysis and allergen detection

**Figure-1:**
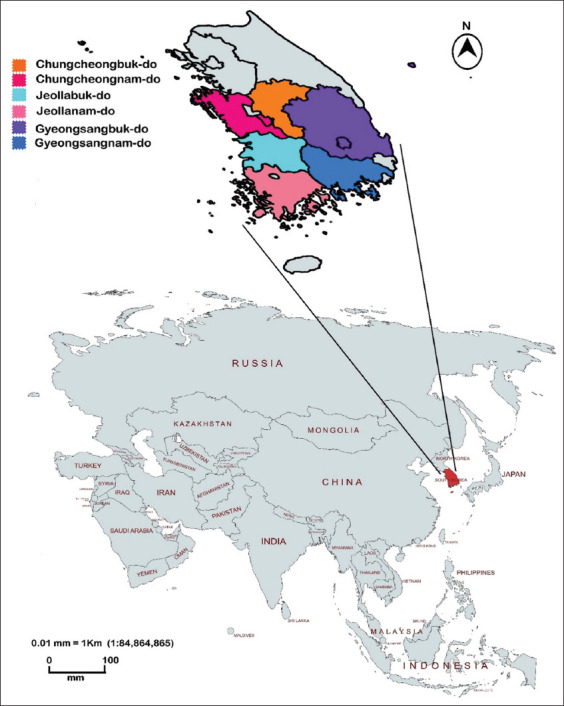
Map of the study area [Source: MAPCHART.NET].

Serum samples were used in this study for the detection of allergen-specific IgEs in dogs with CAD. The Anitia Canine IgE multiple panel ELISA kit (Proteome Tech Inc., Seoul, South Korea [Figure-1]) was employed according to the manufacturer’s instructions (http://www.proteometech.com/en/product/#caninelge).

The allergens tested were grouped as follows: (1) dairy, yeast, and egg, including baker’s yeast, egg white, egg yolk, milk, and cheddar/gouda; (2) grains, including wheat, corn, rice, pea, and soybean; (3) vegetables, fruits, and peanuts, including carrot, potato, sweet potato, pumpkin, tomato, apple, and peanut; (4) meat, including pork, beef, duck, chicken, lamb, turkey, and red deer; (5) seafood, including crab/shrimp, codfish, tuna, salmon, and mackerel; (6) animals, including cat epithelium, wool, and feather mix; (7) molds, including *Penicillium notatum*, *Cladosporium herbarum*, *A. fumigatus*, *Candida albicans*, *Alternaria alternata*, and *Malassezia pachydermatis*; (8) insects and mites, including flea, cockroach, *D*. *pteronyssinus*, *Dermatophagoides farinae*, *Acarus*, and *Tyrophagus putrescentiae*; and (9) tree, including alder/birch, hazel, maple leaf sycamore, willow/cottonwood, oak, white pine, acacia, and white ash; and weed and grass, including common ragweed, plantain, mugwort, sheep’s sorrel, Bermuda grass, orchard/timothy, ryegrass, and cultivated rye.

### Statistical analysis

Statistical analysis was performed using GraphPad Prism 5.03 software (GraphPad Software Inc., San Diego, CA, USA). Independent risk factors for sensitization to each animal allergen were evaluated using Chi-squared tests. Prevalence is expressed as a percentage.

## Results

### Provinces

**Figure-2:**
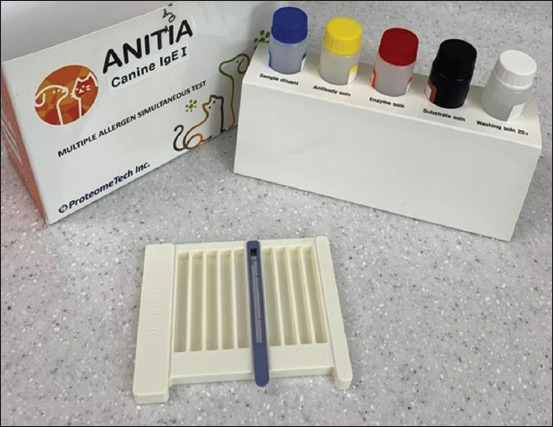
Anitia canine immunoglobulin E multiple panels enzyme-linked immunoassay kit used in the study.

This result indicates that dogs are highly sensitive to allergens from the end of summer through autumn until the beginning of winter. Moreover, the proportion of cases presented for allergy testing were 26.4%, 25.5%, 29.2%, 0.9%, 44.7%, and 4.6% in the Chungcheongbuk-do, Chungcheongnam-do, Jeollabuk-do, Jeollanam-do, Gyeongsangbuk-do, and Gyeongsangnam-do provinces, respectively. Figure-2 shows the map of the study area.

### Breed

Table-1 shows the frequency of different breeds of dogs brought for the allergy test. The Maltese, Bichon Frise, and Poodle breeds had the highest frequency, while the Welsh Corgi, Chihuahua, Husky, Spitz, Italian, and Greyhound types had the lowest percentage of dogs with CAD during the allergy test, representing only 0.6 percent each. The Maltese breed represented 32.3% of all cases. These data indicated that the Maltese breed is the most sensitive to allergens.

### Sex and age

The overall number of cases examined was 103 (54.5%) males and 86 (45.5%) females, the age represented 24.5%, 43.2%, and 54.2% of ≤1-year-olds, 2–3-year-olds, and ≥3-years-olds, respectively. This finding reinforces that male and elderly dogs with CAD symptoms are the most subjected to allergy.

### Percentages of cases with CAD during an allergy test

**Figure-3:**
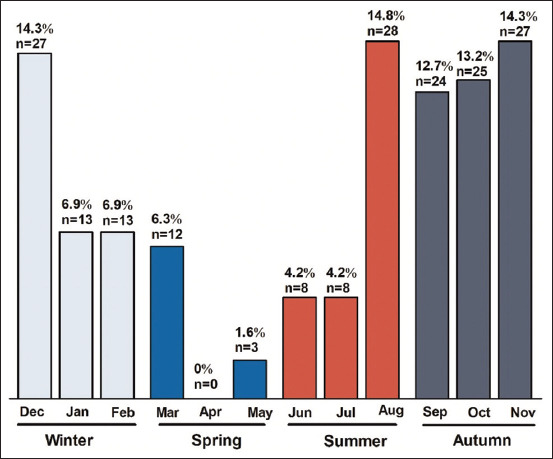
Percentage and the accumulative number of cases tested per month/season. Cumulative number = n.

Figure-3 shows the overall number and percentage of dogs with CAD signs presented to the clinics for IgE-specific allergy test each month. The largest percentages were recorded in August, November, and December with 14.8% (n=28), 14.3% (n=27), and 14.3% (n=27), respectively. The lowest percentages, conversely, were 0% (n=20), 1.6% (n=3), and 4.2% (n=8) in April, May, and June/July, consecutively.

### Mold, insect, and mite allergens

**Figure-4:**
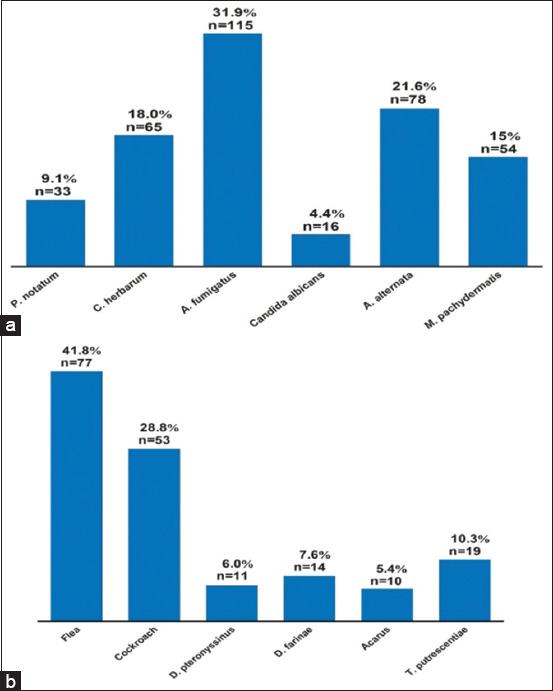
Frequency and the cumulative number of mold, insect, and mild allergens. (a) Mold. (b) Insect and mite. Cumulative number = n.

As shown in Figure-4a, for mold, *A. fumigatus* was the most frequently detected allergen (31.9%), while *C. albicans* (4.4%) was the least detected. As shown in Figure-4b, fleas and acarus were found in 41.8% and 5.4% of the analyzed samples, respectively. This data implies that mold may cause canine sensitization.

### Meat, seafood, and animal part allergens

**Figure-5:**
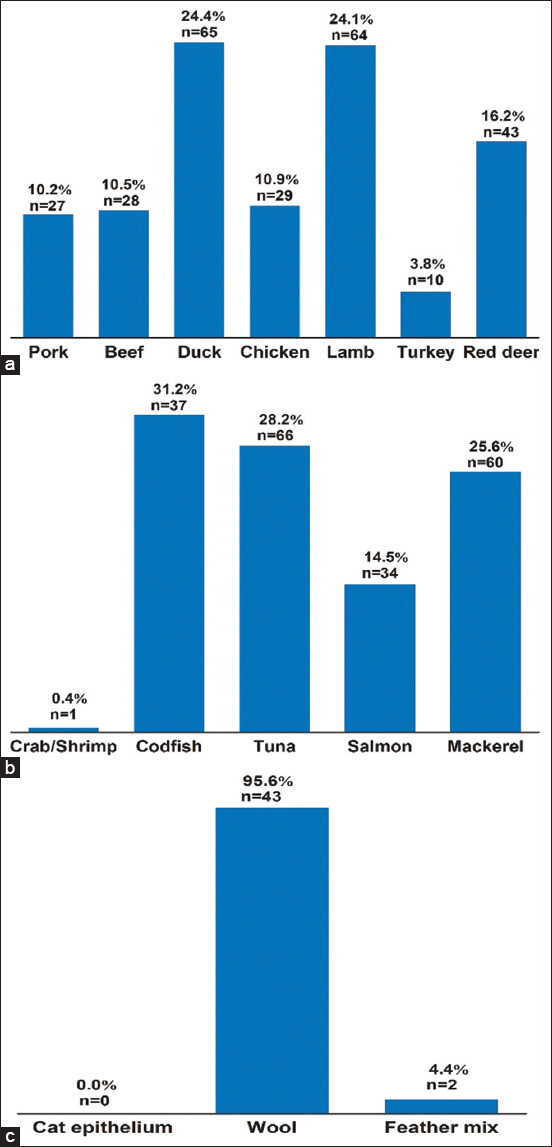
Percentage and cumulative number of meat, seafood, and animal parts allergens. (a) Meat. (b) Seafood. (c) Animal. Cumulative number = n.

Several types of IgE-mediated meat sensitivity were investigated. Figure-5a shows that, whereas IgE-specific allergens in duck and lamb meat had the highest detection rates (24.4% and 24.1%, respectively), dogs with CAD had the lowest sensitivity to turkey meat (3.8%). Furthermore, seafood may cause sensitization in dogs. Figure-5b shows that, with the exception of crab and shrimp, the frequency of IgE-specific allergens in codfish, tuna, salmon, and mackerel ranged from 14.5% to 31.2%. Figure-5c shows that dogs appear to be sensitized only to wool, with a 95.6 % incidence compared to feather mix (4.4 %) and cat epithelium (no instances observed).

### Dairy, yeast, egg, and grain allergens

**Figure-6:**
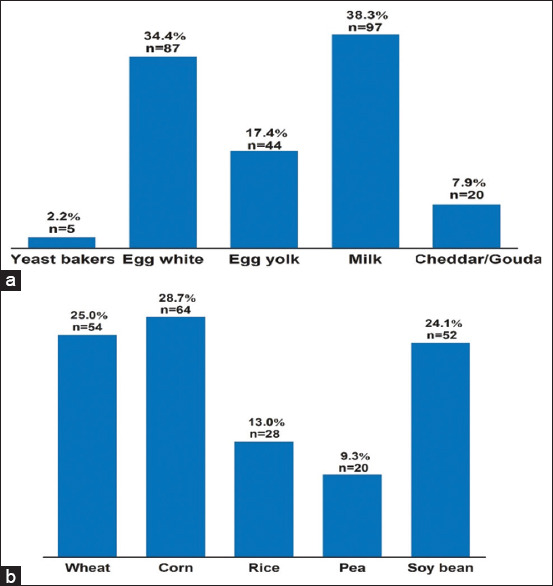
Rate and cumulative number of dairy, yeast, and egg and grain allergens. (a) Dairy, yeast, and egg. (b) Grain. Cumulative number = n.

Among dairy, yeast, egg, milk (38.3 %) and egg white (34.4 %) were the most frequently occurring allergens in dogs diagnosed with CAD, but baker’s yeast (2.2%) remained the least allergenic food to produce sensitization. However, all grains studied are sensitizers based on IgE-specific allergen test. Corn, wheat, and soybeans all had at least a % frequency, according to Figure-6b.

### Trees, vegetables, weeds, and grass allergens

**Figure-7:**
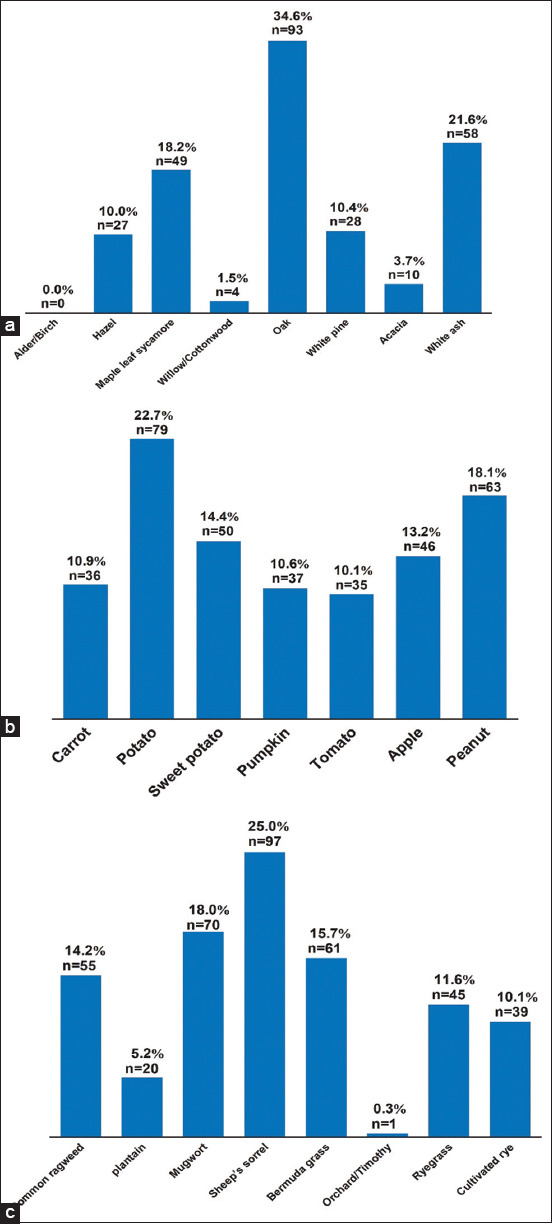
Percentage and aggregated number of trees, vegetables, weeds, and grass allergens. (a) Trees. (b) Vegetables. (c) Weeds and grass. Cumulative number = n.

Plant allergens are commonly found in dogs environment [17]. As indicated in Figure-7a, the most often detected allergens were oak (34.6 %), white ash (21.6 %), and maple leaf of sycamore (18.2 %), whereas willow/cottonwood, white pine, hazel, and acacia remained within the range of 1.5 % -10 % each. Alder/birch is not an allergy inducer in any of the tested samples. Furthermore, all vegetables examined exhibited considerable frequencies of at least 10.1% in tomato, with higher frequency in potato (22.7%) and peanut (18.1%), as shown in Figure-7b. Outdoor weeds and grass can potentially be allergies for pets. According to Figure-7c, allergens frequently identified in dogs include IgE-specific allergens of sheep’s sorrel, mugwort, common ragweed, and Bermuda grass, which account for 72.7% of the cases, whereas IgE-specific allergens of plantain, ryegrass, and cultivated rye are only found in 27.3% of the samples analyzed. This finding suggests that allergens can be consumed through food, such as vegetables, or through interaction with the surrounding environment, such as weeds and grass.

## Discussion

This study used serological IgE-specific allergens to explore allergens in dogs with CAD between January and December 2021. CAD was shown to be prevalent in male canines older than three years old during the fall season. The Maltese breed is the most impacted, with the most cases documented in the Gyeongsangbuk-do province. The incidence of IgE-specific allergens in dogs with CAD is addressed in this paper. The identification of allergens responsible for CAD is critical for treatment and management [18].

Our results (Figure 3) demonstrate that the percentages of total allergens are high from the end of summer to the beginning of winter, then decline until April, when no cases are presented for allergy testing. Following that, it is regularly high, peaking in August. From August through December, animals are most likely to be impacted by CAD. According to recent studies, CAD is more common in the dry season (1.6 times more common than in the rainy season), which has been associated with high temperatures and humidity [1], and in the fall season [19]. Our findings corroborate these reports. Although another study revealed that low temperatures and cloudy weather were associated with an increased risk of atopic dermatitis in humans in Denmark [20], our findings contradict this. This dissimilarity, however, can be attributable to a variation in species and location.

The findings (Table 2) show a significant association between the season and the incidence and percentage of allergens found. In contrast, while discrepancies in the data given imply that age, sex, and province are not risk factors for the development of CAD, our findings are consistent with a previously published study that revealed that age and gender had no effect on the prevalence of allergen sensitization [21]. According to this study and earlier publications [22,23], elderly Maltese, Poodle, Bichon Frise, Shih Tzu, and Pomeranian dogs are the most prone to CAD

**Table-2:** Potential factors analysis of the occurrence of IgE-specific allergens sensitization.

Categories	Number of animals (%)	Times of allergens detected (%)	Degree of freedom	Chai-square	p-value
Gender					
Male	103 (54.5)	1530 (54.8)	1	0.006	0.94
Female	86 (45.5)	1263 (45.2)			
Age					
≤1	88 (24.5)	601 (21.5)	2	0.888	0.64
2–3	67 (43.2)	1290 (46.2)			
≥–	84 (54.2)	54.2 (54.2)			
Season					
Winter	53 (28)	533 (19.1)	3	61.5	0.001
Spring	15 (7.9)	59 (2.1)			
Summer	44 (23.3)	369 (13.2)			
Autumn	77 (40.7)	1832 (65.6)			
Province					
Chungcheongbuk-do	38 (20.1)	504 (18.1)	5	6.599	0.252
Chungcheongnam-do	37 (19.4)	577 (20.7)			
Jeollabuk-do	42 (22.2)	585 (20.9)			
Jeollanam-do	1 (0.7)	20 (0.7)			
Gyeongsangbuk-do	64 (34.0)	1007 (36.0)			
Gyeongsangnam-do	7 (3.5)	98 (3.5)			

p-values were estimated based on the Chi-square test.* P<*0.05 was considered statistically significant. IgE=Immunoglobulin E

Mold, insects, and mites all play important roles in dog sensitization. The findings (Figure 4a, b) show that the most common allergens detected are *A. fumigatus*, *A. alternata, M. Pachydermatis, C. herbarum*, flea, and cockroach (18.0%–41.8%, each). Numerous reports found similar results. For example, Di and coworkers identified diverse types of allergens in dogs using IgE-specific allergen kits in Italy and found that mold allergens detected represented 24%–44.8%, with insects and mites representing 8.9%–13.3% [6]. Furthermore, a recent study found that *D. farinae* and *D. pteronyssinus* are the primary causes of sensitization in humans [24]. The grocery store or the surrounding environment might be the source of these allergies [25].

In this study, 84 % were attributable to tuna, mackerel, and codfish allergies as well as in previous studies [26,27], seafood and animals are substantial sources of allergens for dogs. Interestingly, crab/shrimp is unlikely to cause allergies (Figure 5). Crab/shrimp allergies were linked to only 0.4% of seafood allergies in our results, which is considered the least common among tested seafood IgE-specific allergens, as well as in a previous study [28]. The high rates can be ascribed to the large amounts of tuna, mackerel, and codfish provided to dogs, which are incorporated by food producers for dog palatability. Despite the fact that wool accounted for 95.4% of the occurrences in the animal parts, cat epithelium was not an allergen to the dogs in the study [29,30].

According to our findings (Figure 6a), milk is the most allergenic dairy, yeast, and egg, product (38.3%). Cow’s milk is the most common allergen in dogs, according to similar findings [31,32]. The greatest allergy levels were found in corn (28.7%) and wheat (25.0%), with peas revealing a 9.3 % allergen level (Figure 6b). Several studies [25,26] have found a significant incidence of sensitization to wheat and maize. Sensitization to meat was high in our research and numerous previously published findings (duck 24.4% and lamb 24.1%) and low in turkey (3.8%) [23,33]. These publications, however, demonstrated a high incidence of turkey meat (97%) as an allergy in dogs, which contradicts our findings (Figure 5). This might be attributed to the nature of the dog’s diet.

Trees, weeds, and grass also contribute to sensitization [34]. Our findings (Figure-7) revealed that oak (34.6%), white ash (21.6%), maple leaf sycamore (18.2), sheep’s sorrel (25.0%), mugwort (18.0%), Bermuda grass (15.7%), and common weed (14.2%) are the major allergens detected. In comparison with international studies, tree, grass, and weed pollen represent 26%, 4%, and 47% of outdoor allergens [35], and a recent case study showed that a total of 687 asthmatic children were assessed and pollen sensitivity was found in 29% of children aged 12 to 24 months [36]. It is difficult to compare prior research since tree and grass allergies in dogs with CAD are rarely studied.

This report encourages doctors to discover prevalent allergens in their clients’ environments to enhance the well-being of sensitized animals. Serological samples taken on a regular basis can be examined for this purpose by utilizing IgE-specific allergens kits at home or at a diagnostic center. Such research might benefit both animals and humans who are allergic to environmental allergens.

## Limitations

This study has a number of strengths and weaknesses. Despite the enormous number of allergens evaluated and different dog breeds in six provinces, the methodology reported here has some shortcomings. First, neither a skin test nor a qualitative ELISA were performed because this study aimed to employ serological assays due to their simplicity and multiplicity. Second, information on the remaining provinces in Korea is scarce, possibly due to their remote position from our center. Further collaboration is thus planned. Other limitations, such as cumulative allergen frequencies dependent on age, gender, and breed, were not examined. Finally, understanding the feed composition of the animals would allow us to link our results with the various feeds of the animals.

## Conclusion

This study aimed to detect IgE-specific allergens in the serum of dogs suffering from CAD and to determine their occurrence based on season, breed, age, and sex using ELISA kits. Mold, mites, vegetables, grains, weeds, and grass are among the allergens that are frequently detected. Based on our findings, we believe that comparable kits for diagnosing human allergies may be developed. Following that, a customized food plan for each dog may be created to eliminate allergy exposure. Dog models can be used as experimental animals to find allergens in the environment, allowing humans who have been exposed to similar settings to be assessed.

## Authors’ Contributions

GA, YP, and HO: Designed the study. GA, JiC, and JO: Performed the experiment. GA, HO, YP, and JC: Drafted and revised the manuscript. All authors have read and approved the final manuscript.
